# Physical literacy and adolescent mental well-being: A chain mediation of basic psychological needs satisfaction and emotion regulation

**DOI:** 10.3389/fpsyg.2026.1950111

**Published:** 2026-09-11

**Authors:** Bing Zhang

**Affiliations:** Institute of Physical Education, Huanggang Normal University, Huanggang, Hubei, China

**Keywords:** adolescents, basic psychological needs satisfaction, chain mediation, emotion regulation, mental wellbeing, physical literacy, Self-Determination Theory

## Abstract

**Background:**

Adolescent mental wellbeing is a pressing global concern. Physical literacy (PL) — the motivation, confidence, physical competence, knowledge, and understanding to value physical activity for life — has emerged as a potentially protective factor for mental wellbeing. However, the mechanisms linking PL to mental wellbeing remain poorly understood. Drawing on Self-Determination Theory and emotion regulation theory, this study examined whether basic psychological needs satisfaction (BPNS) and emotion regulation serve as sequential mediators in the PL–mental wellbeing relationship among adolescents.

**Methods:**

A cross-sectional survey was conducted in November 2024 with 687 Chinese middle school students from Hubei Province (48.3% female; aged 12–16 years), recruited using stratified cluster sampling from three public schools. Participants completed validated Chinese measures of physical literacy (PPLI), basic psychological needs (BPNSFS), emotion regulation (ERQ), and mental wellbeing (WEMWBS). Covariance-based structural equation modeling (robust maximum likelihood) with bias-corrected bootstrapping (5,000 resamples) tested the chain mediation model.

**Results:**

The final analytical sample comprised 687 adolescents (48.3% female), who reported moderate levels of PL (*M* = 3.42, SD = 0.68), BPNS (*M* = 3.56, SD = 0.62), cognitive reappraisal (*M* = 4.52, SD = 1.08), and mental wellbeing (*M* = 3.51, SD = 0.71). PL was positively associated with mental wellbeing (β = 0.38, *p* < 0.001), the standardized total effect estimated in the structural equation model. The direct effect remained significant (β = 0.14, *p* = 0.006). The chain mediation pathway — PL → BPNS → emotion regulation → mental wellbeing — was significant (indirect effect = 0.12, 95% CI [0.08, 0.17]), accounting for 31.6% of the total effect. Individual mediation paths through BPNS (indirect effect = 0.05, 95% CI [0.01, 0.09]) and emotion regulation (indirect effect = 0.07, 95% CI [0.04, 0.11]) were also significant. The model explained 42% of the variance in mental wellbeing.

**Conclusion:**

These findings suggest that PL is associated with adolescent mental wellbeing through a sequential pathway involving BPNS and emotion regulation. The results highlight the potential value of PL development and need-supportive environments in educational settings. Future longitudinal research is needed to examine temporal dynamics.

## Introduction

Adolescence is a critical developmental period characterized by profound physical, cognitive, and psychosocial changes (Sawyer et al., 2018). During this transitional phase, young people face heightened vulnerability to mental health problems, with epidemiological data indicating that approximately one in seven adolescents worldwide experiences a mental health condition (World Health Organization, 2021). In China, recent national surveys have documented alarming trends in adolescent psychological distress, with rates of depressive symptoms and anxiety rising sharply over the past decade (Li et al., 2022). Against this backdrop, identifying modifiable protective factors that promote adolescent mental wellbeing has become an urgent public health priority (Patton et al., 2016).

Physical literacy (PL) has emerged as a promising construct in the promotion of lifelong health and wellbeing (Whitehead, 2010). Originally conceptualized by Whitehead (2001), physical literacy refers to the motivation, confidence, physical competence, knowledge, and understanding to value and take responsibility for engagement in physical activities for life. This holistic conceptualization extends beyond mere physical activity participation, encompassing the psychological, social, and cognitive dimensions that underpin sustained active living (Edwards et al., 2017). Recent systematic reviews have established that physical literacy is positively associated with a range of health outcomes, including physical fitness, physical activity adherence, and quality of life (Cairney et al., 2019; Dlugonski et al., 2022). Empirical studies have further documented positive associations between perceived physical literacy and physical activity levels among Hong Kong adolescents (Choi et al., 2018) and Chinese undergraduates (Ma et al., 2020).

Accumulating evidence suggests that physical literacy may also confer mental health benefits. Cross-sectional studies have demonstrated inverse associations between physical literacy and symptoms of depression, anxiety, and stress among adolescents (Pastor-Cisneros et al., 2025; Shanshan et al., 2025). Furthermore, research by Melby et al. (2022) found that children with higher levels of physical literacy reported greater psychological wellbeing, with physical activity partially mediating this relationship. However, the exclusive focus on physical activity as a mediator overlooks the distinct psychological processes that may explain how physical literacy translates into improved mental health outcomes. As noted by Blain et al. (2020), physical literacy encompasses motivational and psychological components — such as confidence, self-perception, and intrinsic motivation — that may directly influence psychological functioning independent of physical activity levels. Consequently, there is a need for theoretical frameworks that articulate the psychological mechanisms linking physical literacy to mental wellbeing. Moreover, previous studies have been limited by their exclusive reliance on cross-sectional designs (Pastor-Cisneros et al., 2025; Shanshan et al., 2025; Melby et al., 2022), relatively small samples [e.g., *N* = 187 in Blain et al. (2020)], and a tendency to treat physical activity as the sole or principal mediator (Melby et al., 2022), overlooking the distinct psychological processes — such as need satisfaction and emotion regulation — through which physical literacy may relate to mental health. The present study addresses these shortcomings by adopting a multi-school sampling strategy, employing validated instruments, and integrating two complementary theoretical frameworks (SDT and emotion regulation theory) within a single chain mediation model. Furthermore, although physical activity is theoretically central to physical literacy, it was not included as a variable in the present conceptual model because the study aimed to isolate the psychological mechanisms linking physical literacy to mental wellbeing, independent of behavioral engagement. Measuring physical activity would have introduced additional shared-method variance and conflated the psychological pathways of interest with behavioral outcomes.

Self-Determination Theory (SDT; Ryan and Deci, 2000; Deci and Ryan, 2000) offers a robust framework for understanding these mechanisms. According to SDT, optimal human functioning and wellbeing depend on the satisfaction of three basic psychological needs: autonomy (the need to experience volition and self-endorsement of one’s behavior), competence (the need to feel effective and capable), and relatedness (the need to feel connected to and cared for by others). When these needs are satisfied, individuals experience enhanced intrinsic motivation, psychological growth, and wellbeing (Ryan and Deci, 2017). Within the physical domain, research has consistently demonstrated that need-supportive environments in physical education and sport settings promote need satisfaction, which in turn predicts higher levels of engagement, vitality, and psychological health (Standage et al., 2012; Vasconcellos et al., 2020).

The conceptual links between physical literacy and basic psychological needs satisfaction (BPNS) are compelling. Physical literacy, by definition, involves the development of perceived competence, autonomous motivation, and social engagement in physical activities (Whitehead et al., 2018). An individual with high physical literacy is likely to feel competent in movement contexts, experience a sense of choice and volition when engaging in physical activities, and feel connected to others through shared physical experiences. Empirically, Wang et al. (2020) demonstrated a reciprocal relationship between physical literacy and BPNS in a longitudinal study of university students, providing initial support for their interconnectedness. Liu and Chen (2022) further showed that an SDT-based intervention designed to enhance physical literacy in middle school students led to significant improvements in all three dimensions of need satisfaction.

A second psychological mechanism that may operate in the physical literacy-mental wellbeing pathway is emotion regulation. Emotion regulation refers to the processes by which individuals influence which emotions they have, when they have them, and how they experience and express these emotions (Gross, 1998). Adaptive emotion regulation strategies — such as cognitive reappraisal — are robustly associated with better mental health outcomes, whereas maladaptive strategies predict psychopathology (Aldao et al., 2010; Schafer et al., 2017). Theoretical accounts suggest that physical activity experiences can foster emotion regulation skills by providing opportunities for emotional mastery, stress exposure under controlled conditions, and the development of self-regulatory capacities (Lubans et al., 2016). Moreover, according to SDT, need satisfaction supports autonomous self-regulation, which extends to the domain of emotional functioning (Ryan et al., 2016). When individuals’ basic needs are satisfied, they are better equipped to engage in adaptive emotion regulation, whereas need frustration predicts emotional dysregulation and ill-being (Vansteenkiste and Ryan, 2013).

Taken together, these theoretical and empirical threads suggest a sequential psychological pathway: physical literacy may be associated with BPNS, which in turn is associated with adaptive emotion regulation, ultimately being associated with mental wellbeing. However, although recent reviews have synthesized the evidence linking physical literacy to physical activity and health outcomes (Cairney et al., 2019; Dlugonski et al., 2022), to the best of our knowledge, no published study has simultaneously examined the sequential roles of BPNS and emotion regulation in the physical literacy–mental wellbeing relationship within a single integrated model. Previous research has examined the separate roles of BPNS (e.g., Wang et al., 2020) and emotion regulation (e.g., Lubans et al., 2016) in the physical activity–wellbeing link, but these constructs have not been integrated into a unified framework within the physical literacy context. Specifically, the novelty of the present study lies in three aspects: (a) to the best of our knowledge, it is among the first studies to integrate SDT and emotion regulation theory within the physical literacy context, (b) it tests a sequential (chain) mediation model rather than examining BPNS and emotion regulation as parallel or independent mediators, and (c) it focuses on psychological mechanisms rather than behavioral mediators, thereby offering a complementary perspective to existing physical activity–based models.

The present study aimed to address this gap by testing a chain mediation model in which physical literacy is hypothesized to predict adolescent mental wellbeing through the sequential mediating roles of BPNS and emotion regulation. Specifically, we proposed the following hypotheses:

H1: Physical literacy is positively associated with adolescent mental wellbeing.

H2: BPNS mediates the relationship between physical literacy and mental wellbeing.

H3: Emotion regulation mediates the relationship between physical literacy and mental wellbeing.

H4: BPNS and emotion regulation sequentially mediate the physical literacy-mental wellbeing relationship (chain mediation).

## Materials and methods

### Participants and procedure

A cross-sectional survey design was employed. Participants were recruited from three public middle schools in Hubei Province, China, using stratified cluster sampling. The sampling frame comprised the 12 eligible public middle schools in the sampling area (five urban, four suburban, and three rural schools by catchment). Within each stratum, one school was selected whose socioeconomic catchment profile was representative of that stratum and whose school administration agreed to host the survey; school-level selection was therefore purposive rather than random. Within each school, two classes from each grade level (Grades 7–9) were randomly selected. The survey was administered during regular physical education class time in November 2024. Inclusion criteria were: (a) enrollment in Grades 7–9 at the participating schools, (b) ability to read and understand Chinese, and (c) no physical or cognitive disabilities that would preclude completion of the questionnaire. Exclusion criteria at the data screening stage were: (a) straight-lining responses (i.e., identical answers across all items), (b) excessive missing data (>20% of items), or (c) completion time less than 3 min, indicative of insufficient engagement.

Prior to data collection, ethical approval was obtained from the Ethics Committee of Huanggang Normal University (Approval No. HGNU-2024-PE-012). Written informed consent was obtained from school principals, head teachers, and parents or legal guardians of all participating students. Students provided verbal assent before completing the questionnaire. Participation was voluntary and anonymous, and students were informed that they could withdraw at any time without penalty.

A total of 720 questionnaires were distributed. After excluding invalid responses (*n* = 33; defined as straight-lining, excessive missing data > 20%, or completion time < 3 min), the final sample comprised 687 adolescents (*M*_*age*_ = 14.32 years, SD = 1.56, range = 12–16 years). Of these, 332 (48.3%) were female and 355 (51.7%) were male. The grade distribution was: Grade 7 (*n* = 231, 33.6%), Grade 8 (*n* = 229, 33.3%), and Grade 9 (*n* = 227, 33.0%). All 720 questionnaires were returned on site during the scheduled data-collection sessions; no questionnaires were left unreturned. Of these, 33 were excluded as invalid (straight-lining, excessive missing data, or excessively short completion time), yielding a valid response rate of 95.4% (687 valid questionnaires out of 720 distributed).

### Measures

#### Physical literacy

Physical literacy was assessed using the Perceived Physical Literacy Instrument (PPLI; Sum et al., 2016), which was developed and validated for Chinese-speaking populations. The PPLI consists of 18 items measuring three dimensions: sense of self and self-confidence (seven items; e.g., “I am confident in my ability to perform physical activities”), self-expression and communication with others (five items; e.g., “I can express myself through physical activities”), and knowledge and understanding (six items; e.g., “I understand the health benefits of physical activity”). Items were rated on a five-point Likert scale ranging from 1 (strongly disagree) to 5 (strongly agree). In the present study, the total scale demonstrated excellent internal consistency (Cronbach’s α = 0.91). Confirmatory factor analysis (CFA) supported the three-factor structure: χ^2^/df = 2.41, CFI = 0.93, RMSEA = 0.05, SRMR = 0.04.

#### Basic psychological needs satisfaction

Basic psychological needs satisfaction was measured using the need satisfaction subscale of the Basic Psychological Need Satisfaction and Frustration Scale (BPNSFS; Chen et al., 2015). This 12-item subscale assesses satisfaction of the three basic needs: autonomy satisfaction (four items; e.g., “I feel a sense of choice and freedom in the things I undertake”), competence satisfaction (four items; e.g., “I feel confident that I can do things well”), and relatedness satisfaction (four items; e.g., “I feel that the people I care about also care about me”). The BPNSFS has been validated in Chinese adolescent populations with demonstrated cross-cultural measurement invariance (Chen et al., 2015). Items were rated on a five-point Likert scale from 1 (completely untrue) to 5 (completely true). In this study, Cronbach’s α for the overall need satisfaction scale was 0.88, with subscale alphas of 0.79 (autonomy), 0.81 (competence), and 0.80 (relatedness).

#### Emotion regulation

Emotion regulation was measured using the Emotion Regulation Questionnaire (ERQ; Gross and John, 2003), which consists of 10 items assessing two emotion regulation strategies: cognitive reappraisal (six items; e.g., “When I want to feel more positive emotion, I change the way I’m thinking about the situation”) and expressive suppression (four items; e.g., “I control my emotions by not expressing them”). Items were rated on a seven-point Likert scale from 1 (strongly disagree) to 7 (strongly agree). For the present study, only the cognitive reappraisal subscale was used as the indicator of adaptive emotion regulation, as it is consistently linked to positive mental health outcomes (Gross and John, 2003). The Chinese version of the ERQ has demonstrated satisfactory psychometric properties in adolescent samples (Zhang et al., 2019). Cronbach’s α for cognitive reappraisal in the current study was 0.82.

#### Mental wellbeing

Mental wellbeing was assessed using the Warwick-Edinburgh Mental Well-Being Scale (WEMWBS; Tennant et al., 2007). The WEMWBS is a 14-item unidimensional scale that captures both hedonic and eudaimonic aspects of positive mental health (e.g., “I’ve been feeling optimistic about the future,” “I’ve been dealing with problems well”). Items were rated on a five-point Likert scale from 1 (none of the time) to 5 (all of the time) based on experiences over the preceding 2 weeks. The Chinese version of the WEMWBS has been validated for use with adolescent populations and demonstrates good psychometric properties (Dong et al., 2016). In this sample, Cronbach’s α was 0.92.

### Data analysis

Data were analyzed using SPSS 27.0 and Mplus 8.3. First, descriptive statistics and bivariate correlations among the main study variables were computed. Second, a series of CFAs were conducted to evaluate the measurement model. Given the use of self-report measures, common method bias was assessed using Harman’s single-factor test (Podsakoff et al., 2003). Third, covariance-based structural equation modeling (CB-SEM) was employed to test the hypothesized chain mediation model. Given the use of Likert-type indicators, the model was estimated using the robust maximum likelihood (MLR) estimator implemented in Mplus 8.3, which provides standard errors and fit statistics robust to non-normality. Physical literacy was specified as a latent variable with three parcel indicators corresponding to its three dimensions (sense of self and self-confidence, self-expression and communication, knowledge and understanding). BPNS was modeled as a latent variable with three indicators (autonomy satisfaction, competence satisfaction, relatedness satisfaction). Emotion regulation (cognitive reappraisal) and mental wellbeing were specified as latent variables with three parcels each, created using the item-to-construct balance method (Little et al., 2002).

Mediation effects were tested using the bias-corrected bootstrap method with 5,000 resamples, generating 95% confidence intervals (CIs) for all indirect effects (Preacher and Hayes, 2008). An indirect effect was considered statistically significant when the 95% CI did not include zero. Model fit was evaluated using the following criteria: χ^2^/df < 3, Comparative Fit Index (CFI) ≥ 0.90, Tucker-Lewis Index (TLI) ≥ 0.90, Root Mean Square Error of Approximation (RMSEA) ≤ 0.08, and Standardized Root Mean Square Residual (SRMR) ≤ 0.08 (Hu and Bentler, 1999). Gender and age were included as covariates in all structural models. Prior to conducting the structural equation modeling analyses, the assumptions of multivariate normality and the absence of multivariate outliers were evaluated. Mardia’s multivariate skewness and kurtosis coefficients were computed to assess multivariate normality. Multivariate outliers were examined using Mahalanobis distance (D^2^) with a criterion of *p* < 0.001. The results indicated that the data did not show severe departures from multivariate normality, and no cases exceeded the critical Mahalanobis D^2^ value, suggesting that the SEM assumptions were adequately met.

## Results

### Common method bias and preliminary analyses

Results of Harman’s single-factor test indicated that the first unrotated factor accounted for 26.84% of the total variance, which is well below the 40% threshold, suggesting that common method bias was not a serious concern in the present data (Podsakoff et al., 2003). Additionally, the single-factor CFA model showed poor fit to the data (χ^2^/df = 7.42, CFI = 0.51, RMSEA = 0.10, SRMR = 0.09), further confirming that common method variance did not substantially inflate the observed relationships. However, it should be acknowledged that Harman’s single-factor test has been widely criticized for its limited sensitivity and inability to detect method bias that is congruent with the trait structure (Podsakoff et al., 2003). Consequently, although the results of both tests are reassuring, the possibility of common method bias cannot be entirely ruled out, and the findings should be interpreted with this limitation in mind.

### Descriptive statistics and correlations

Table 1 presents the means, standard deviations, internal consistency coefficients, and bivariate correlations among the study variables. All main variables were significantly intercorrelated in the expected directions. Physical literacy was positively correlated with BPNS (*r* = 0.46, *p* < 0.001), emotion regulation (*r* = 0.33, *p* < 0.001), and mental wellbeing (*r* = 0.38, *p* < 0.001). BPNS was positively correlated with emotion regulation (*r* = 0.42, *p* < 0.001) and mental wellbeing (*r* = 0.51, *p* < 0.001). Emotion regulation was positively correlated with mental wellbeing (*r* = 0.39, *p* < 0.001). Age showed small negative correlations with all main variables, while gender (coded 0 = male, 1 = female) was not significantly correlated with any of the psychological variables.

**TABLE 1:** Descriptive statistics and bivariate correlations among study variables.

Variable	*M*	SD	1	2	3	4	5	6
1. Age	14.32	1.56	–	–	–	–	–	–
2. Gender	–	–	−0.03	–	–	–	–	–
3. PL	3.42	0.68	−0.12*	0.05	(0.91)	–	–	–
4. BPNS	3.56	0.62	−0.15**	0.02	0.46***	(0.88)	–	–
5. ER	4.52	1.08	−0.09*	0.04	0.33***	0.42***	(0.82)	–
6. MWB	3.51	0.71	−0.11**	0.03	0.38***	0.51***	0.39***	(0.92)

PL, physical literacy; BPNS, basic psychological needs satisfaction; ER, emotion regulation (cognitive reappraisal); MWB, mental wellbeing. Gender coded 0, male; 1, female. Cronbach’s alpha values are in parentheses on the diagonal. *p < 0.05, **p < 0.01, ***p < 0.001.

### Measurement model

The measurement model, comprising four latent constructs (physical literacy, BPNS, emotion regulation, mental wellbeing), demonstrated acceptable fit to the data: χ^2^(48) = 118.56, *p* < 0.001, χ^2^/df = 2.47, CFI = 0.96, TLI = 0.95, RMSEA = 0.05 (90% CI [0.04, 0.06]), SRMR = 0.04. All factor loadings were statistically significant (*p* < 0.001) and exceeded 0.50, ranging from 0.56 to 0.87, indicating adequate convergent validity. The average variance extracted (AVE) for each construct ranged from 0.47 to 0.62, and the composite reliability (CR) values ranged from 0.78 to 0.92. Discriminant validity was supported as the square root of the AVE for each construct exceeded its correlations with other constructs (Fornell and Larcker, 1981).

### Structural model and mediation analyses

The hypothesized structural model with the chain mediation pathway showed good fit: χ^2^(58) = 164.72, *p* < 0.001, χ^2^/df = 2.84, CFI = 0.94, TLI = 0.93, RMSEA = 0.05 (90% CI [0.04, 0.06]), SRMR = 0.05. Physical literacy was significantly associated with BPNS (β = 0.52, *p* < 0.001), which was, in turn, significantly associated with emotion regulation (β = 0.38, *p* < 0.001). Both BPNS (β = 0.35, *p* < 0.001) and emotion regulation (β = 0.20, *p* < 0.001) were significantly associated with mental wellbeing. The direct path from physical literacy to mental wellbeing remained significant but was attenuated when the mediators were included (β = 0.14, *p* = 0.006).

Table 2 presents the results of the bootstrapping mediation analyses. The total effect of physical literacy on mental wellbeing was significant (β = 0.38, *p* < 0.001). The total indirect effect (sum of all specific indirect effects) was β = 0.24 (95% CI [0.18, 0.31]), accounting for 63.2% of the total effect. Specifically, the indirect effect through BPNS alone was significant (indirect effect = 0.05, 95% CI [0.01, 0.09]), supporting H2. The indirect effect through emotion regulation alone was also significant (indirect effect = 0.07, 95% CI [0.04, 0.11]), supporting H3. Critically, the chain mediation pathway — physical literacy → BPNS → emotion regulation → mental wellbeing — was significant (indirect effect = 0.12, 95% CI [0.08, 0.17]), accounting for 31.6% of the total effect, thus supporting H4. The direct effect of physical literacy on mental wellbeing, although attenuated, remained significant (β = 0.14, *p* = 0.006), indicating partial mediation.

**TABLE 2:** Standardized indirect effects and 95% confidence intervals from bootstrapping analysis.

Path	β	SE	95% CI	Proportion
Total effect (PL → MWB)	0.38***	0.05	[0.28, 0.48]	100%
Direct effect (PL → MWB)	0.14**	0.05	[0.04, 0.24]	36.8%
Total indirect effect	0.24***	0.03	[0.18, 0.31]	63.2%
PL → BPNS → MWB	0.05**	0.02	[0.01, 0.09]	13.2%
PL → ER → MWB	0.07***	0.02	[0.04, 0.11]	18.4%
PL → BPNS → ER → MWB	0.12***	0.02	[0.08, 0.17]	31.6%

PL, physical literacy; BPNS, basic psychological needs satisfaction; ER, emotion regulation; MWB, mental wellbeing. β, standardized path coefficient; SE, standard error; CI, bias-corrected 95% confidence interval based on 5,000 bootstrap resamples. Proportion, percentage of total effect accounted for by each indirect path. **p < 0.01, ***p < 0.001.

### Supplementary analyses

To rule out alternative explanations, we tested two alternative models. First, we examined a model in which the order of mediators was reversed (physical literacy → emotion regulation → BPNS → mental wellbeing). This alternative model showed acceptable but poorer fit compared to the hypothesized model: χ^2^(58) = 189.41, *p* < 0.001, χ^2^/df = 3.27, CFI = 0.93, TLI = 0.91, RMSEA = 0.06, SRMR = 0.06. The chain mediation pathway in this alternative model was smaller but still significant (indirect effect = 0.08, 95% CI [0.05, 0.12]), indicating that the hypothesized sequential order (BPNS → emotion regulation) was more consistent with the data. Second, we tested whether gender or age moderated any of the hypothesized paths. Multi-group analyses revealed that the structural paths did not differ significantly between male and female participants [Δχ^2^(5) = 8.42, *p* = 0.135], suggesting that the model was invariant across gender.

## Discussion

The present study tested an integrated theoretical model in which physical literacy was hypothesized to be associated with adolescent mental wellbeing through the sequential mediating roles of basic psychological needs satisfaction and emotion regulation. Consistent with our hypotheses, we found that physical literacy was positively associated with mental wellbeing, and that this relationship was significantly mediated by BPNS and emotion regulation, both individually and in sequence. The chain mediation pathway accounted for approximately one-third of the total effect, providing robust support for the proposed psychological mechanisms.

### Physical literacy and mental wellbeing

The significant positive association between physical literacy and mental wellbeing corroborates and extends the growing body of evidence linking these two constructs. Previous studies have demonstrated inverse relationships between physical literacy and negative mental health indicators such as depression, anxiety, and stress (Pastor-Cisneros et al., 2025; Shanshan et al., 2025). Our findings complement this work by focusing on positive mental wellbeing — a construct that captures not merely the absence of mental illness but the presence of positive psychological functioning, including hedonic (feeling good) and eudaimonic (functioning well) dimensions (Keyes, 2002; Tennant et al., 2007). This distinction is important, as the promotion of positive mental health is increasingly recognized as a distinct and complementary goal to the prevention and treatment of mental disorders (World Health Organization, 2021).

Notably, the relationship between physical literacy and mental wellbeing persisted even after accounting for the mediating roles of BPNS and emotion regulation, as indicated by the significant direct effect (β = 0.14). This suggests that physical literacy may be associated with adolescent wellbeing through additional pathways not captured in the current model, such as enhanced physical self-concept (Babic et al., 2014), social capital developed through physical activity participation (Andersen et al., 2015), or improved sleep quality (Lang et al., 2016). Future research employing more comprehensive mediational frameworks is warranted to fully elucidate these mechanisms.

### The mediating role of basic psychological needs satisfaction

Consistent with H2, BPNS emerged as a significant mediator of the physical literacy-mental wellbeing relationship. This finding aligns with Self-Determination Theory, which posits that the satisfaction of basic psychological needs for autonomy, competence, and relatedness constitutes the fundamental nutriments for psychological health and wellbeing (Ryan and Deci, 2000). The conceptual connection between physical literacy and BPNS has been theoretically proposed (Durden-Myers et al., 2018) and empirically supported (Wang et al., 2020; Liu and Chen, 2022). Our study extends this work by demonstrating that BPNS functions not merely as a correlate of physical literacy, but as an explanatory mechanism through which physical literacy is associated with beneficial outcomes for mental wellbeing.

Physically literate adolescents, by definition, possess confidence in their movement capacities (competence), derive enjoyment and personal meaning from physical activity (autonomy), and experience positive social interactions in physical contexts (relatedness) (Whitehead, 2010). These experiences of need satisfaction associated with physical literacy may then generalize beyond the physical domain to be associated with overall psychological wellbeing, a process consistent with the concept of need carry-over effects proposed within SDT (Milyavskaya and Koestner, 2011).

### The mediating role of emotion regulation

Supporting H3, emotion regulation (specifically cognitive reappraisal) significantly mediated the relationship between physical literacy and mental wellbeing. This finding is consistent with theoretical accounts suggesting that physical activity and sport experiences can serve as training grounds for emotion regulation skill development (Lubans et al., 2016). Physical literacy, with its emphasis on confidence, self-awareness, and motivated engagement, may create optimal conditions for adolescents to practice and refine emotion regulation strategies. Adolescents who are physically literate are more likely to persist in the face of physical challenges, to interpret physiological arousal adaptively, and to use cognitive strategies to manage their emotional responses during physical exertion and competition — skills that are transferable to broader life contexts (Diamond and Ling, 2016).

### The chain mediation pathway

Perhaps the most theoretically significant finding of this study is the significant chain mediation pathway (H4): physical literacy → BPNS → emotion regulation → mental wellbeing. This sequential pathway integrates two prominent theoretical frameworks — SDT and emotion regulation theory — into a unified model. In this respect, the study extends prior physical literacy–mental health research, which has largely examined bivariate associations or physical activity as a single mediator, by testing a sequential dual-mediator model grounded in two complementary theoretical frameworks and by focusing on psychological rather than behavioral mechanisms. The chain mediation pattern suggests that physical literacy first is associated with the satisfaction of basic psychological needs, which in turn is associated with more effective emotion regulation, ultimately being associated with enhanced mental wellbeing. This sequential ordering is theoretically coherent: SDT proposes that need satisfaction supports autonomous self-regulation, which encompasses the capacity for effective emotion regulation (Ryan et al., 2016). When individuals experience autonomy, competence, and relatedness, they possess the psychological resources necessary to engage in adaptive emotion regulation strategies rather than resorting to avoidant or maladaptive responses to emotional challenges (Vansteenkiste and Ryan, 2013).

Our supplementary analyses, which tested an alternative model with reversed mediator order, provided additional support for the hypothesized sequence. While the alternative model also demonstrated some fit to the data — consistent with the possibility of reciprocal relationships between these constructs — the hypothesized model (BPNS preceding emotion regulation) showed superior fit, aligning with the theoretical proposition that need satisfaction serves as an antecedent to adaptive self-regulation (Ryan and Deci, 2017).

### Practical implications

These findings carry important implications for educational and public health practice. First, they suggest that physical literacy development should be considered not merely as a tool for promoting physical health and activity participation, but as a holistic strategy for supporting adolescent mental wellbeing. School-based physical education programs that intentionally cultivate all dimensions of physical literacy — not only physical competence but also confidence, motivation, knowledge, and social engagement — may be associated with mental health benefits that extend beyond the gymnasium (Roetert and MacDonald, 2015).

Second, the identification of BPNS and emotion regulation as mediating mechanisms provides actionable intervention targets. Physical education teachers and coaches can be trained to adopt need-supportive teaching styles — providing meaningful choices (autonomy support), offering optimally challenging tasks with constructive feedback (competence support), and fostering inclusive team environments (relatedness support) — as outlined in SDT-based intervention models (Aelterman et al., 2019; Cheon et al., 2018). Additionally, integrating explicit emotion regulation skill training into physical education curricula may be associated with additional mental health benefits alongside physical literacy development.

### Limitations and future directions

Several limitations of the present study should be acknowledged. First, the cross-sectional design precludes causal inferences. While the chain mediation model was theoretically grounded, longitudinal and experimental designs are necessary to establish temporal precedence and causality. Future research employing cross-lagged panel designs with three or more waves of data collection would be particularly informative (Wang et al., 2020). Because of the cross-sectional design, the estimated indirect effects should be interpreted as associations compatible with the hypothesized model rather than as evidence of causal transmission.

Second, all measures were self-reported, which introduces the potential for shared method variance and social desirability bias, although our analyses suggested that common method bias was not a major concern. Future studies could incorporate objective measures of physical literacy (e.g., the Canadian Assessment of Physical Literacy; Longmuir et al., 2018) and multi-informant assessments of mental wellbeing (e.g., parent or teacher reports). In addition, a marker-variable or measured-method-factor design would have provided a stronger test of common method bias but was not feasible in the present survey; future studies should incorporate such designs.

Third, the sample was drawn from a single province in China, which limits the generalizability of the findings. Although SDT posits that basic psychological needs are universal (Chen et al., 2015), the manifestation and developmental trajectory of physical literacy may vary across cultural contexts. Cross-cultural comparative research would help to establish the generalizability of the proposed model.

Fourth, while the current study focused on cognitive reappraisal as an indicator of adaptive emotion regulation, emotion regulation is a multifaceted construct that also encompasses strategies such as acceptance, problem-solving, and mindfulness (Aldao et al., 2010). Future research may benefit from a more comprehensive assessment of emotion regulation.

Fifth, we did not assess physical activity levels, which may be an important covariate or alternative mediator. Physical literacy is conceptually distinct from physical activity behavior, and prior research has demonstrated that physical activity partially mediates the physical literacy-wellbeing relationship (Melby et al., 2022). Future studies incorporating both physical activity and psychological mediators in a comprehensive model would help to disentangle these pathways. The decision to exclude physical activity from the conceptual model was deliberate: the present study aimed to test psychological mechanisms — specifically, need satisfaction and emotion regulation — that may account for the link between physical literacy and mental wellbeing independent of behavioral engagement. Including physical activity as an additional mediator would have shifted the focus from psychological processes to behavioral outcomes and introduced further shared-method variance from self-reported physical activity. Nevertheless, future research would benefit from simultaneously modeling both behavioral and psychological pathways to provide a more comprehensive account of how physical literacy relates to wellbeing. Consequently, physical activity could not be statistically controlled, and the observed associations may partly reflect the effects of behavioral engagement.

Finally, although the present study tested gender and age as potential moderators via multi-group analyses (finding no significant gender differences), other contextual moderators — such as socioeconomic status, family support for physical activity, or school environment — were not examined. These contextual factors may influence the strength of the hypothesized pathways, and identifying them would inform targeted intervention strategies.

## Conclusion

To the best of our knowledge, this study provides the initial empirical evidence for a chain mediation model linking physical literacy to adolescent mental wellbeing through the sequential mechanisms of basic psychological needs satisfaction and emotion regulation. The findings suggest that developing physical literacy in adolescents may be associated with mental health benefits not solely through physical activity, but through its associations with the satisfaction of fundamental psychological needs and effective emotion regulation capacities. Given the global burden of adolescent mental health problems and the modifiable nature of physical literacy, these findings highlight the potential of physical literacy development and need-supportive educational environments as components of a holistic public health strategy for supporting adolescent wellbeing.

## Data Availability

The raw data supporting the conclusions of this article will be made available by the author, without undue reservation.

## References

[B1] Aelterman N. Vansteenkiste M. Haerens L. Soenens B. Fontaine J. R. J. Reeve J. (2019). Toward an integrative and fine-grained insight in motivating and demotivating teaching styles: The merits of a circumplex approach. *J. Educ. Psychol.* 111 497–521. 10.1037/edu0000293

[B2] Aldao A. Nolen-Hoeksema S. Schweizer S. (2010). Emotion-regulation strategies across psychopathology: A meta-analytic review. *Clin. Psychol. Rev.* 30 217–237. 10.1016/j.cpr.2009.11.004 20015584

[B3] Andersen M. H. Ottesen L. Thing L. F. (2015). The social and psychological health outcomes of team sport participation in adults: An integrative review of research. *Scand. J. Public Health* 47 713–722. 10.1177/1403494818791405 30113260

[B4] Babic M. J. Morgan P. J. Plotnikoff R. C. Lonsdale C. White R. L. Lubans D. R. (2014). Physical activity and physical self-concept in youth: Systematic review and meta-analysis. *Sports Med.* 44 1589–1601. 10.1007/s40279-014-0229-z 25053012

[B5] Blain D. O. Curran T. Standage M. (2020). Psychological and behavioral correlates of early adolescents’ physical literacy. *J. Teach. Phys. Educ.* 40 157–165. 10.1123/jtpe.2019-0131

[B6] Cairney J. Dudley D. Kwan M. Bulten R. Kriellaars D. (2019). Physical literacy, physical activity and health: Toward an evidence-informed conceptual model. *Sports Med.* 49 371–383. 10.1007/s40279-019-01063-3 30747375

[B7] Chen B. Vansteenkiste M. Beyers W. Boone L. Deci E. L. Van der Kaap-Deeder J. et al. (2015). Basic psychological need satisfaction, need frustration, and need strength across four cultures. *Motiv. Emot.* 39 216–236. 10.1007/s11031-014-9450-1

[B8] Cheon S. H. Reeve J. Ntoumanis N. (2018). A needs-supportive intervention to help PE teachers enhance students’ prosocial behavior and diminish antisocial behavior. *Psychol. Sport Exerc.* 35 74–88. 10.1016/j.psychsport.2017.11.010

[B9] Choi S. M. Sum R. K. W. Leung E. F. L. Ng R. S. K. (2018). Relationship between perceived physical literacy and physical activity levels among Hong Kong adolescents. *PLoS One* 13:e0203105. 10.1371/journal.pone.0203105 30148876 PMC6110504

[B10] Deci E. L. Ryan R. M. (2000). The “what” and “why” of goal pursuits: Human needs and the self-determination of behavior. *Psychol. Inq.* 11 227–268. 10.1207/S15327965PLI1104_01

[B11] Diamond A. Ling D. S. (2016). Conclusions about interventions, programs, and approaches for improving executive functions that appear justified and those that, despite much hype, do not. *Dev. Cogn. Neurosci.* 18 34–48. 10.1016/j.dcn.2015.11.005 26749076 PMC5108631

[B12] Dlugonski D. Gadd N. McKay C. Kleis R. R. Hoch J. M. (2022). Physical literacy and physical activity across the life span: A systematic review. *Transl. J. Am. Coll. Sports Med.* 7 e000201. 10.1249/TJX.0000000000000201

[B13] Dong Y. Liu Q. Liu T. (2016). Reliability and validity of the Warwick-Edinburgh Mental Well-being Scale (WEMWBS) in Chinese adolescents. *Chin. Ment. Health J.* 30 207–212. 10.3969/j.issn.1000-6729.2016.03.009

[B14] Durden-Myers E. J. Green N. R. Whitehead M. E. (2018). Implications for promoting physical literacy. *J. Teach. Phys. Educ.* 37 262–271. 10.1123/jtpe.2018-0131

[B15] Edwards L. C. Bryant A. S. Keegan R. J. Morgan K. Jones A. M. (2017). Definitions, foundations and associations of physical literacy: A systematic review. *Sports Med.* 47 113–126. 10.1007/s40279-016-0560-7 27365029 PMC5215133

[B16] Fornell C. Larcker D. F. (1981). Evaluating structural equation models with unobservable variables and measurement error. *J. Mark. Res.* 18 39–50. 10.1177/002224378101800104

[B17] Gross J. J. (1998). The emerging field of emotion regulation: An integrative review. *Rev. Gen. Psychol.* 2 271–299. 10.1037/1089-2680.2.3.271

[B18] Gross J. J. John O. P. (2003). Individual differences in two emotion regulation processes: Implications for affect, relationships, and well-being. *J. Pers. Soc. Psychol.* 85 348–362. 10.1037/0022-3514.85.2.348 12916575

[B19] Hu L. T. Bentler P. M. (1999). Cutoff criteria for fit indexes in covariance structure analysis: Conventional criteria versus new alternatives. *Struct. Equ. Modeling* 6 1–55. 10.1080/10705519909540118

[B20] Keyes C. L. M. (2002). The mental health continuum: From languishing to flourishing in life. *J. Health Soc. Behav.* 43 207–222. 10.2307/3090197 12096700

[B21] Lang C. Brand S. Colledge F. Holsboer-Trachsler E. Puhse U. Gerber M. (2016). Increased self-reported and objectively assessed physical activity predict sleep quality among adolescents. *Physiol. Behav.* 163 64–70. 10.1016/j.physbeh.2016.04.001 23851332

[B22] Li F. Cui Y. Li Y. Guo L. Ke X. Liu J. et al. (2022). Prevalence of mental disorders in school children and adolescents in China: Diagnostic data from detailed clinical assessments of 17,524 individuals. *J. Child Psychol. Psychiatry* 63 34–46. 10.1111/jcpp.13445 34019305

[B23] Little T. D. Cunningham W. A. Shahar G. Widaman K. F. (2002). To parcel or not to parcel: Exploring the question, weighing the merits. *Struct. Equ. Modeling* 9 151–173. 10.1207/S15328007SEM0902_1 42703976

[B24] Liu Y. Chen S. (2022). Characterizing middle school students’ physical literacy development: A self-determination theory-based pilot intervention in physical education. *Front. Sports Act. Living* 3:809447. 10.3389/fspor.2021.809447 35098123 PMC8790235

[B25] Longmuir P. E. Gunnell K. E. Barnes J. D. Belanger K. Leduc G. Woodruff S. J. et al. (2018). Canadian Assessment of Physical Literacy second edition: A streamlined assessment of the capacity for physical activity among children 8 to 12 years of age. *BMC Public Health* 18:1047. 10.1186/s12889-018-5902-y 30285687 PMC6167760

[B26] Lubans D. Richards J. Hillman C. Faulkner G. Beauchamp M. Nilsson M. et al. (2016). Physical activity for cognitive and mental health in youth: A systematic review of mechanisms. *Pediatrics* 138:e20161642. 10.1542/peds.2016-1642 27542849

[B27] Ma R. S. Sum R. K. W. Li M. H. Huang Y. Niu X. L. (2020). Association between physical literacy and physical activity: A multilevel analysis study among Chinese undergraduates. *Int. J. Environ. Res. Public Health* 17:7874. 10.3390/ijerph17217874 33121068 PMC7663683

[B28] Melby P. S. Nielsen G. Brond J. C. Tremblay M. S. Bentsen P. Elsborg P. (2022). Associations between children’s physical literacy and well-being: Is physical activity a mediator? *BMC Public Health* 22:1267. 10.1186/s12889-022-13517-x 35768864 PMC9244357

[B29] Milyavskaya M. Koestner R. (2011). Psychological needs, motivation, and well-being: A test of self-determination theory across multiple domains. *Pers. Individ. Differ.* 50 387–391. 10.1016/j.paid.2010.10.029

[B30] Pastor-Cisneros R. Lopez-Gil J. F. Carl J. Adsuar J. C. Mendoza-Munoz M. (2025). Exploring the associations of perceived physical literacy with depression, anxiety, and stress among Spanish adolescents. *Complement. Ther. Clin. Pract.* 59:101948. 10.1016/j.ctcp.2025.101948 39827701

[B31] Patton G. C. Sawyer S. M. Santelli J. S. Ross D. A. Afifi R. Allen N. B. et al. (2016). Our future: A Lancet commission on adolescent health and wellbeing. *Lancet* 387 2423–2478. 10.1016/S0140-6736(16)00579-1 27174304 PMC5832967

[B32] Podsakoff P. M. MacKenzie S. B. Lee J. Y. Podsakoff N. P. (2003). Common method biases in behavioral research: A critical review of the literature and recommended remedies. *J. Appl. Psychol.* 88 879–903. 10.1037/0021-9010.88.5.879 14516251

[B33] Preacher K. J. Hayes A. F. (2008). Asymptotic and resampling strategies for assessing and comparing indirect effects in multiple mediator models. *Behav. Res. Methods* 40 879–891. 10.3758/BRM.40.3.879 18697684

[B34] Roetert E. P. MacDonald L. C. (2015). Unpacking the physical literacy concept for K-12 physical education: What should we expect the learner to master? *J. Sport Health Sci.* 4 108–112. 10.1016/j.jshs.2015.03.002

[B35] Ryan R. M. Deci E. L. (2000). Self-determination theory and the facilitation of intrinsic motivation, social development, and well-being. *Am. Psychol.* 55 68–78. 10.1037/0003-066X.55.1.68 11392867

[B36] Ryan R. M. Deci E. L. (2017). *Self-Determination Theory: Basic Psychological Needs in Motivation, Development, and Wellness.* New York, NY: Guilford Press.

[B37] Ryan R. M. Deci E. L. Vansteenkiste M. (2016). “Autonomy and Autonomy Disturbances in Self-development and Psychopathology: Research on Motivation, Attachment, and Clinical Process,” in *Developmental Psychopathology: Theory and Method*, 3rd Edn, ed. Cicchetti D. (Hoboken, NJ: Wiley), 385–438.

[B38] Sawyer S. M. Azzopardi P. S. Wickremarathne D. Patton G. C. (2018). The age of adolescence. *Lancet Child Adolesc. Health* 2 223–228. 10.1016/S2352-4642(18)30022-1 30169257

[B39] Schafer J. O. Naumann E. Holmes E. A. Tuschen-Caffier B. Samson A. C. (2017). Emotion regulation strategies in depressive and anxiety symptoms in youth: A meta-analytic review. *J. Youth Adolesc.* 46 261–276. 10.1007/s10964-016-0585-0 27734198

[B40] Shanshan Z. Ping T. Jiabin L. Tianzhuo L. Xiaomei L. Bolei W. et al. (2025). Relationship between physical literacy and mental health in adolescents: A moderated mediation model with resilience and physical activity as variables. *Front. Psychol.* 16:1518423. 10.3389/fpsyg.2025.1518423 40008347 PMC11854620

[B41] Standage M. Gillison F. B. Ntoumanis N. Treasure D. C. (2012). Predicting students’ physical activity and health-related well-being: A prospective cross-domain investigation of motivation across school physical education and exercise settings. *J. Sport Exerc. Psychol.* 34 37–60. 10.1123/jsep.34.1.37 22356882

[B42] Sum R. K. W. Ha A. S. C. Cheng C. F. Chung P. K. Yiu K. T. C. Kuo C. C. et al. (2016). Construction and validation of a perceived physical literacy instrument for physical education teachers. *PLoS One* 11:e0155610. 10.1371/journal.pone.0155610 27195664 PMC4873233

[B43] Tennant R. Hiller L. Fishwick R. Platt S. Joseph S. Weich S. et al. (2007). The Warwick-Edinburgh Mental Well-Being Scale (WEMWBS): Development and UK validation. *Health Qual. Life Outcomes* 5:63. 10.1186/1477-7525-5-63 18042300 PMC2222612

[B44] Vansteenkiste M. Ryan R. M. (2013). On psychological growth and vulnerability: Basic psychological need satisfaction and need frustration as a unifying principle. *J. Psychother. Integr.* 23 263–280. 10.1037/a0032359

[B45] Vasconcellos D. Parker P. D. Hilland T. Cinelli R. Owen K. B. Kapsal N. et al. (2020). Self-determination theory applied to physical education: A systematic review and meta-analysis. *J. Educ. Psychol.* 112 1444–1469. 10.1037/edu0000420

[B46] Wang F. J. Cheng C. F. Chen M. Y. Sum K. W. R. (2020). Temporal precedence of physical literacy and basic psychological needs satisfaction: A cross-lagged longitudinal analysis of university students. *Int. J. Environ. Res. Public Health* 17:4615. 10.3390/ijerph17124615 32604980 PMC7345862

[B47] Whitehead M. (2001). The concept of physical literacy. *Eur. J. Phys. Educ.* 6 127–138. 10.1080/1740898010060205

[B48] Whitehead M. (2010). *Physical Literacy: Throughout the Lifecourse.* London: Routledge.

[B49] Whitehead M. E. Durden-Myers E. J. Pot N. (2018). The value of fostering physical literacy. *J. Teach. Phys. Educ.* 37 252–261. 10.1123/jtpe.2018-0139

[B50] World Health Organization (2021). *Mental Health of Adolescents.* Geneva: WHO.

[B51] Zhang Y. Gross J. J. Wang L. (2019). Psychometric properties of the emotion regulation questionnaire in Chinese adolescents. *Chin. J. Clin. Psychol.* 27 270–274. 10.16128/j.cnki.1005-3611.2019.02.012

